# Feasibility of Multiple Micro-Particle Trapping—A Simulation Study

**DOI:** 10.3390/s150304958

**Published:** 2015-02-27

**Authors:** Yanyan Yu, Weibao Qiu, Bernard Chiu, Lei Sun

**Affiliations:** 1 Department of Electronic Engineering, City University of Hong Kong, Hong Kong, China; E-Mails: yanyanyu2-c@my.cityu.edu (Y.Y.); bcychiu@cityu.edu.hk (B.C.); 2 Paul C. Lauterbur Research Center for Biomedical Imaging, Institute of Biomedical and Health Engineering, Shenzhen Institutes of Advanced Technology, Chinese Academy of Sciences, Shenzhen 518055, China; E-Mail: wb.qiu@siat.ac.cn; 3 Interdisciplinary Division of Biomedical Engineering, the Hong Kong Polytechnic University, Hong Kong, China

**Keywords:** acoustic tweezers, multiple trapping, phased array transducer

## Abstract

Both optical tweezers and acoustic tweezers have been demonstrated for trapping small particles in diverse biomedical applications. Compared to the optical tweezers, acoustic tweezers have deeper penetration, lower intensity, and are more useful in light opaque media. These advantages enable the potential utility of acoustic tweezers in biological science. Since the first demonstration of acoustic tweezers, various applications have required the trapping of not only one, but more particles simultaneously in both the axial and lateral direction. In this research, a method is proposed to create multiple trapping patterns, to prove the feasibility of trapping micro-particles. It has potential ability to electronically control the location and movement of the particles in real-time. A multiple-focus acoustic field can be generated by controlling the excitation of the transducer elements. The pressure and intensity of the field are obtained by modeling phased array transducer. Moreover, scattering force and gradient force at various positions are also evaluated to analyze their relative components to the effect of the acoustic tweezers. Besides, the axial and lateral radiation force and the trapping trajectory are computed based on ray acoustic approach. The results obtained demonstrate that the acoustic tweezers are capable of multiple trapping in both the axial and lateral directions.

## 1. Introduction

Optical tweezers have been applied to biology and physical research after they were first introduced in 1986 [1], and have been applied in manipulating micro/nano particles such as bacteria, cells, molecular motors, and single molecules [2]. Although optical tweezers have excellent precision as demonstrated in many applications [3], they have several limitations. Optical tweezers are largely limited to transparent media. The trapping ability is significantly reduced in the presence of opaque media, which scatters and attenuates the optical ray on its path to the particles. In addition, optical tweezers are associated with poor penetration in the biotic environment, such as skin and other human tissues. Moreover, previous studies [4,5,6] have demonstrated that optical tweezers can cause physiological damage to biological objects due to laser-induced heating.

Previous studies have also demonstrated the feasibility of manipulating particles by acoustic waves [7,8,9,10,11]. Acoustic tweezers can propagate deeper than optical tweezers in opaque biomedical environments, giving them many advantages in biological applications, such as drug delivery. In contrast with highly focused laser or electron beams, ultrasound has been proven to be safe to biomedical tissues and has been used in diagnostic imaging for over 60 years [12,13]. These two major advantages have driven the development of acoustic tweezers.

Many studies have demonstrated the feasibility of acoustic waves for the non-contact and non-invasive manipulation of microscopic particles. The first report of acoustic tweezers was given by Wu [7] in 1991. A stable force potential well generated by two identical focusing transducers (3.5 MHz) was demonstrated to trap latex particles and clusters of frog eggs. In 1995, Hertz [8] described a 3D trapping mechanism for microscopic particles in a standing wave field produced by two collimated ultrasound transducers. Courtney *et al*. [9] demonstrated that 5 μm polystyrene particles could be trapped and moved in one dimension using a pair of acoustically matched piezoelectric transducers (5.25 MHz) that generate the standing wave field. However, acoustic trappings described above were achieved using acoustic waves produced by multiple transducers; the implementation of a multiple-transducer system is complicated, which involves small trapping spacing between two collimated transducers that restricts its application in one side open configuration.

To address this problem, single-transducer acoustic trapping mechanisms have been proposed. Lee *et al*. [14] applied a highly focused ultrasonic transducer (100 MHz) to generate radiation forces to trap target particle along axial direction in Mie regime (in which particle size is six times larger than wavelength). Kang and Yeh [11] designed an acoustic tweezer based on a four-element planar transducer with low driving frequency (1 MHz). In his simulation, Laguerre-Gaussian model is generated by the four-element planar transducer for confining small particles in Rayleigh regime (in which particles are much smaller than the wavelength).

Since the first demonstration of acoustic tweezers, various potential applications have required the simultaneous trapping of many particles rather than one particle only, including sorting small particles [15], controlling multi-molecular complexes [16], particle-patterning technology [17] *etc*. While many optical tweezers have now been designed with multiple trapping capability [18], to the best of our knowledge, we were the first to describe single-transducer acoustic tweezers that have multiple trapping ability in a three-dimensional setting [19]. In this paper, we further improve the previous system in the area of scattering and gradient force analysis, the effect of acoustic streaming and resistance due to viscosity of medium in trapping performance.

In this paper, we describe a multiple trapping acoustic tweezers with four focal points, each of which can control a single particle. To demonstrate the feasibility of this system, the radiation forces of a particle along and across the beam propagation direction are analyzed in one of the four trapping points. Further, the trapping trajectory along and across the beam propagation directions was determined with the consideration of acoustic streaming effect and the resistance due to the viscosity of water. Scattering force and gradient force components at various positions were also evaluated to analyze their relative contribution to the effect of the multiple trapping acoustical tweezers. We demonstrate that the proposed acoustic tweezer can generate a set of trapping points in different positions.

## 2. Methods

**Figure 1:**
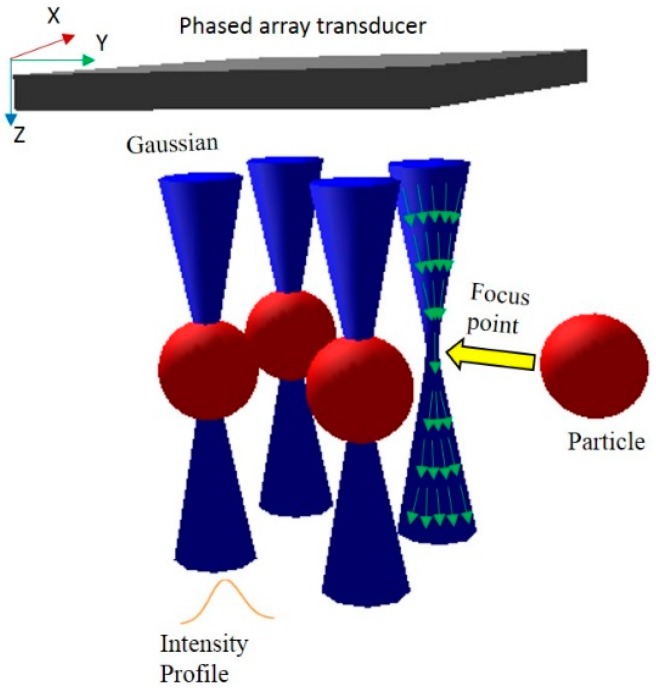
Schematic of the multiple trapping acoustic tweezers. The blue hyperbolic space shows the Gaussian beams generated by a phased array transducer (gray plane). The orange line is the intensity profile of Gaussian beam. The red ball is the objective particle to be trapped by the focal points in the center of beams. Green arrows denote wave propagation direction in Gaussian beam.

The proposed multiple trapping system is based on a multiple-focus field generated by a phased array transducer. Each focal point in this acoustic tweezers can be used to manipulate an individual particle. The schematic of the system is shown as Figure 1. In Section 2.1, we describe the approach to calculate the excitation vector for each phased array element. In Section 2.2, the multiple-focus field patterns are synthesized with continuous excitation from every element in the phased array transducer instead of assumed Gaussian intensity distribution [10].

**Figure 2:**
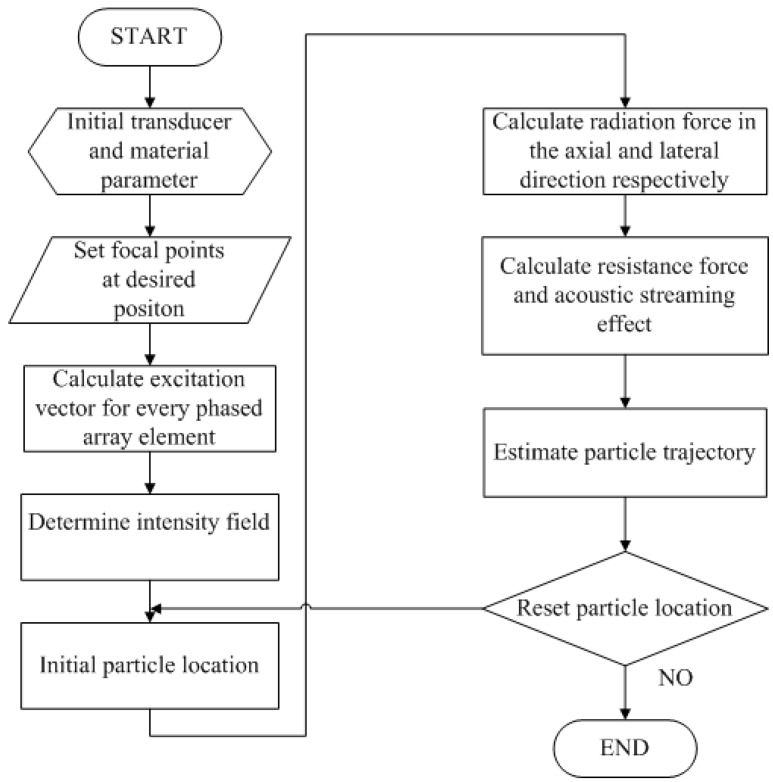
Flow chart for creating the multiple-focus acoustic field and calculating the radiation force.

The theory of acoustic tweezers based on ray acoustic approach is introduced to calculate radiation force on the axial and lateral direction in Section 2.3. Resistance force and acoustic streaming effect are calculated in Section 2.4. The overall mechanism of the multiple trapping acoustic tweezers is outlined in the flow chart shown in Figure 2.

### 2.1. Determine Excitation for Each Phased Array Element

**Figure 3:**
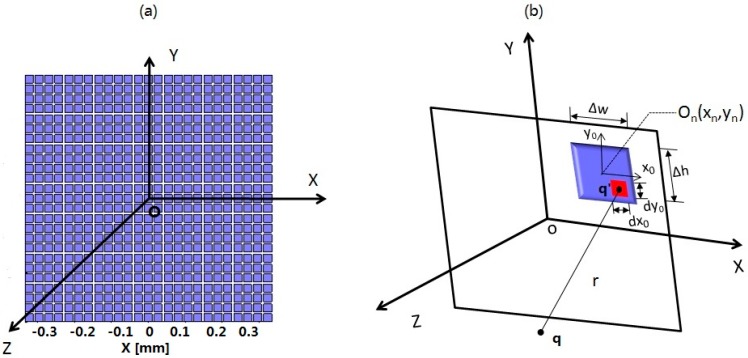
Configuration and geometry for the phased array sensor (**a**) Phased array sensor with 25 × 25 elements; (**b**) Coordinate system for the calculation of the acoustic field.

A 50 MHz two-dimensional (2D) phased array transducer with 25 × 25 elements, which is shown as Figure 3a was used to generate an acoustic field with multiple focal points for multiple trapping. In this section, a method is introduced to calculate the excitation vector of the every array element to generate desired multiple-focus field patterns. A matrix form propagation operator [20] generated by the discretized Rayleigh-Sommerfeld was applied in this work. The acoustic pressure from source point q′ to a target point q in Figure 2a can be expressed as:

(1) $$p\left(q\right)=\frac{\;j\rho_{0}c}{\lambda }\int_{S}u(q\text{′})\frac{e^{j(\omega t-k|q-q\text{′}|)}}{\left|q-q\text{′}\right|}dS$$

where p(q) is acoustic pressure at point *q*, j = $\sqrt{-1}$, $\rho_{0}$ and c are the density and the speed of sound in the medium, respectively, λ is sound wavelength, $e^{j\omega t}$ is time dependence term, ω is acoustic wave frequency, k is acoustic wavenumber, *q* and *q* ′ are, respectively, the target and source points. S denotes the surface of source. u is excitation of the source point. For the phased array we used to generate multiple trapping energy field, Equation (1) can be rewritten as:

(2) $$p\left(q\right)=\frac{\;j\rho_{0}c}{\lambda }\overset{N}{\underset{n=1}{\sum }}u_{n}\int_{S_{n}}u(q\text{′})\frac{e^{j(\omega t-k|q-q{\text{′}}_{n}|)}}{\left|q-q{\text{′}}_{n}\right|}dS_{n}$$

where $S_{\text{n}}$ is the nth element surface, $u_{\text{n}}$ is the excitation of the nth element. $q{\text{′}}_{\text{n}}$ represents source points on the nth element. Suppose the pressure at the M focal spots that used to trap target particle is known, Equation (2) can be rewritten as:

(3) $$p\left(q_{m}\right)=\frac{\;j\rho_{0}c}{\lambda }\sum_{n=1}^{N}u_{n}\int_{S_{n}}\frac{e^{j(\omega t-k|q_{m}-q_{n}^{\text{′}}|)}}{\left|q_{m}-q_{n}^{\text{′}}\right|}dS_{n}\;m=1,2\ldots ,M$$

or in matrix form:

(4) p=HU

where *p* = [$p\left(q_{1}\right)$*,* $\;p\left(q_{2}\right)$*, …,* $\;p\left(q_{m}\right)$] is a vector representing pressure at M focal spots in the multiple trapping energy field. Vector $U=\;\left[u_{1},\;u_{2},\ldots ,\;u_{\text{N}}\right]$ denotes a vector including excitation of N elements. *H* is a matrix denoting acoustic wave forward propagation, its element can be expressed mathematically as:

(5) $$H_{\left(m,n\right)}=\;\frac{\;j\rho_{0}c}{\lambda }\int_{S_{n}}\frac{e^{j(\omega t-k|q_{m}-q_{n}^{\text{′}}|)}}{\left|q_{m}-q_{n}^{\text{′}}\right|}dS_{n}$$

in which $H_{\left(\text{m},\text{n}\right)}$ represents acoustic wave propagation from source space on the nth element to the mth focal spots. In Equation (4), U can be obtained by inverting the propagation matrix using the generalized inverse approach, expressed as:

(6) $$U=H^{\text{*}t}{\left(HH^{\text{*}t}\right)}^{-1}p$$

$H^{*t}$ denotes the pseudoinverse of H, which can be obtained by calculating decomposition value of matrix *H* using SVD.

To increase the array excitation efficiency, an iterative weighting algorithm is applied based on Equation (6):

(7) $$\hat{U}\;=WH^{*t}{\left(HWH^{*t}\right)}^{-1}p$$

where W is an N × N real, positive definite weighting matrix. It could be initialized as an identity matrix first. Then an iterative weighting algorithm [20] is taken to yield W. Finally, given the pressure p at the M focal spots, vector $\hat{U}$, which includes excitation vector of every phased array element that makes specified power at the focal points, can be resolved.

### 2.2. Acoustic Field Modeling

The pressure at a point in the field can be obtained by summarizing all the acoustic pressure from each infinitesimal element. Figure 3b illustrates the definition of the coordinate system and geometry. *Δw* and *Δh* indicate the width and height of the phased array element. *O*_n_, the center of the *n*th array element, is signified by (*x*_n_, *y*_n_) in coordinate system (*x*, *y*, *z*). A secondary coordinate system denoted by (*x*_0_, *y*_0_) has the center of the nth array element as its origin. Every phased array element is divided into infinitesimal elements, each of which acts as a small source. *dx*_0_*dy*_0_ is the surface of the infinitesimal element. For a uniformly moving radiation source with $e^{j\omega t}$ time dependence, the acoustic pressure [21] is given by Equations (8):

(8) $$p\left(x,y,z,t\right)=\frac{\;j\rho_{0}c}{\lambda }\overset{N}{\underset{n=1}{\sum }}{\hat{U}}_{n}e^{j\omega t}\int_{-∆h/2}^{∆h/2}\int_{-∆w/2}^{∆w/2}\frac{1}{r}e^{\left(-jkr\right)}dx_{0}dy_{0}$$

where r represents the distance from the center of infinitesimal element to the observation point in the field, $\rho_{0}$ and c are the density and the speed of sound in the medium, respectively. The vector *p*(*x, y, z, t*) indicates the pressure at observation point (*x*, *y*, *z*) at time *t*. The excitation of the *n*th array element ${\hat{U}}_{n}$ can be obtained by Equation (7). Based on Equation (8), the intensity value *I*(*x*, *y*, *z*, *t*) for each point at time *t* in the field is expressed as [22]:

(9) $$I\left(x,y,z,t\right)=\frac{p\times p\text{*}}{2\rho_{0}c}$$

where p and p* are, acoustic pressure in Equation (8) and its conjugate. $\;\rho_{0}$ and c are the density and the speed of sound in the medium, respectively.

### 2.3. The Theory of Acoustic Tweezers

**Figure 4:**
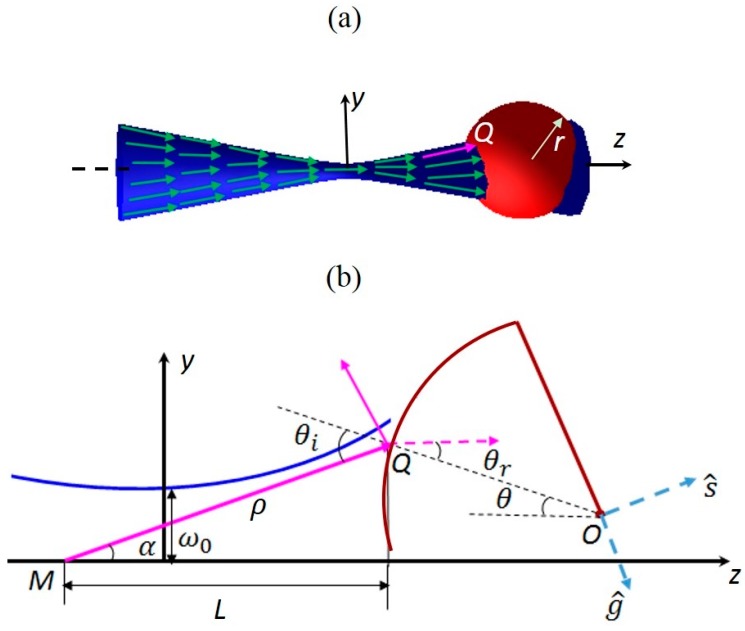
(**a**) An overall view of a single beam acoustic tweezers. Blue hyperbolic space represents the focused acoustic beam. Green vectors denote the wave propagation direction. Pink arrow is an individual incident ray hit on the target particle. Red ball is the target particle; (**b**) 2D zooming geometry for the analysis of the radiation force when a sphere located at the arbitrary location. Blue line represents the acoustic beam. The incident ray assumed from point M with angle *α* to the beam axis *z* hits the spherical particle (red line) at point *Q*. The absolute distance between M and Q is ρ, which is also the radius of the spherical wave front. *L* is the axial distance between M and Q. $\vec{OQ}$ and *z* axis has angle θ to each other. $\theta_{\text{i}}$ and $\theta_{\text{r}}$ indicate the incident angle and refractive angle at the interacting point Q respectively. $\hat{s}$ and $\hat{g}$ are the unit vectors representing the directions of the scattering and the gradient forces. Beam waist size is $\omega_{0}$.

Since acoustic waves possess similar physical properties as optical waves, the theory of calculation of the radiation force for acoustic tweezers is analogous to optical tweezers in the Mie regime, where particle diameters are at least six times larger than the wavelength. Figure 4a shows an overall view of a single Gaussian beam acoustic tweezers and a target particle (red ball) with radius $r_{0}$ suspending in a liquid. The wave propagation direction (green arrows) convergent in front of the trapping point and divergent beyond it.

Figure 4b shows the 2D geometry for the analysis of the radiation force. In a Gaussian distributed beam, an individual incident ray $\vec{MQ}$ with power w strikes a small region on the surface of the particle at point *Q*. According to the law of conservation, the radiation force caused by an individual ray can be decomposed into two orthogonal components known as scattering force component *dF_s_* , which mainly arise from reflection momentum transfer, and gradient force component *dF_g_*, which mainly arise from refraction momentum transfer. Commonly they can be written as [10,23]:

(10) $$dF_{s}=\frac{w\;}{c}q_{s}=\frac{w\;}{c}(1+R\;cos2\theta_{i}-\frac{T^{2}[cos\left(2\theta_{i}\;-\;2\theta_{r}\right)\;+\;R\;cos2\theta_{i}]}{1\;+\;R^{2}\;+\;2R\;cos2\theta_{r}})$$

(11) $$dF_{g}=\frac{w\;}{c}q_{g}=\frac{w\;}{c}(Rsin2\theta_{i}-\frac{T^{2}[sin\left(2\theta_{i}\;-\;2\theta_{r}\right)\;+\;R\;sin2\theta_{i}]}{1\;+\;R^{2\;}+\;2R\;cos2\theta_{r}})$$

where w and *c* represent the average power of the incident ray and the acoustic speed in the medium, respectively. $q_{s}$ and ${\text{q}}_{g}$ are dimensionless fractions of the peak scattering force and gradient force [24] transferred to the sphere by the emergent ray, respectively. $\theta_{i}$ and $\theta_{r}$ are the incident and transmitted angles. R and T are the Fresnel reflection coefficient and transmission coefficient at the surface of the sphere, they are given by:

(12) $$R=|\frac{Z_{2}/cos\theta_{i}-Z_{1}/cos\theta_{r}}{Z_{2}/cos\theta_{i}+Z_{1}/cos\theta_{r}}|$$

(13) T=1−R

where $Z_{1}$ and $Z_{2}$ are the acoustic impedances in the surrounding medium and the particles.Then the radiation force along z direction $dF_{z}$ and along y direction $dF_{y}$ on Figure 4a can be estimated by decomposing *dF_s_* and *dF_g_*, they can be written as Equations (14) and (15):

(14) $$dF_{z}=dF_{sz}+dF_{gz}$$

(15) $$dF_{y}=dF_{sy}+dF_{gy}$$

in which, $dF_{sz}$ and $dF_{gz}$ indicate the z component of the scattering force $dF_{s}$ and gradient force $dF_{g}$, likewise, $dF_{sy}$ and $dF_{gy}$ represent the y component of the scattering force $dF_{s}$ and gradient force $dF_{g}$, respectively. As integrating $dF_{z}$ and $dF_{y}$ from the differential area over which the incident ray hits the particle, it leads to the total force $F_{z}$ and $F_{y}$ produced by the entire beam. Finally, in the Mie regime where particle size is larger than the wavelength of incident ray, the complete formulations of radiation force in both axial and lateral directions can be described as follows:

(16) $$F_{z}=F_{sz}+F_{gz}=\int^{\text{​}}\frac{r^{2}Isin\theta cos\theta_{i}}{c}(q_{s}\vec{s_{z}}+q_{g}\vec{g_{z}})d\theta$$

(17) $$F_{y}=F_{sy}+F_{gy}=\int^{\text{​}}\frac{r^{2}Isin\theta cos\theta_{i}}{c}(q_{s}\vec{s_{y}}+q_{g}\vec{g_{y}})d\theta$$

where c is the speed of sound in the medium; θ is the angle between the beam axis and line $\;\vec{OQ}$. $\theta_{i}$ indicates the incident angle at the interacting point *Q*. $r_{0}$ is the radius of the sphere. Term $\frac{r^{2}Isin\theta cos\theta_{i}}{c}d\theta$ represents an individual ray with power w entering the particle across a differential area. $\hat{s}$ and $\hat{g}$ are the unit vectors representing the directions of the scattering and the gradient forces shown as Figure 4b. Here, they are decomposed into the *y* and *z* directions and denoted as $\left(\vec{s_{\text{z}}},\vec{s_{\text{y}}}\right)$ and $\left(\vec{g_{\text{z}}},\;\vec{g_{\text{y}}}\right)$. These two vectors can be derived as Equations (18)–(21) geometrically:

(18) $$\hat{s}=\;\frac{\vec{MQ}}{\left|MQ\right|}=\left(\vec{s_{z}},\vec{s_{y}}\right)=(\frac{L}{\rho },\frac{Q_{y}}{\rho })$$

(19) $$\hat{g}=\left(g_{z},g_{y}\right)=(\frac{Q_{y}}{\rho },-\frac{L}{\rho })$$

(20) $$L=\pm \sqrt{\rho^{2}-{Q_{y}}^{2}}$$

(21) $$\rho =Q_{z}[1+{\left(\frac{\pi \omega_{0}^{2}}{\lambda Q_{z}}\right)}^{2}]$$

where λ is the wavelength of the incident ray; $Q_{\text{z}}$ and $Q_{\text{y}}$ are coordinates of Q point. $\vec{MQ}$ represents the vector from M to Q. |MQ| is the absolute distance from M to Q. Other signs in Equations (18)–(21) are defined in the Figure 4b.

### 2.4. Resistance Force and Acoustic Streaming Effect 

In the numerical analysis of the object motion, the resistance due to viscosity of medium is also taken into consideration in the form of Stokes’ formula as Equation (21) [25]:

(22) $$F_{r}=-6\pi \mu r_{0}v$$

where *μ* is the viscosity coefficient of medium; $r_{0}$ and *v* are the radius and velocity of the spherical particle, respectively.

Drag force caused by acoustic streaming is also taken into account, since experiments [7] has found that the main disruption in acoustic trapping is the competition force due to acoustic streaming. To investigate the acoustic streaming effect on multiple-trapping acoustic tweezers, the drag force due to acoustic streaming is taken into account by [26]:

(23) $$F_{d}=3\text{πσ}v_{s}\omega_{0}$$

where σ is the dynamic viscosity of medium, $\omega_{0}$ is the beam width. $v_{s}$ is the axial streaming velocity, which can be estimated by the approach proposed by Nowicki [27].

## 3. Results and Discussion

The theoretical method to create acoustic field and radiation force for the multiple trapping has been introduced in Section 2. In this section, the results will be shown to demonstrate the feasibility of this multiple trapping acoustic tweezers model. A 2D phased array transducer consisted of 625 elements with kerf width 5 μm, and pitch size 27 μm is used to generate and control the multiple-focus acoustic field as shown in Figure 3a. The simulation parameters including the detailed characteristics of the phased array and material parameters are summarized in Table 1. Here a lipid or fat sphere was chosen as the object, supposing its homogeneous refractive index is higher than that of the surrounding medium.

**Table 1:** Simulation parameters.

Elements Number of Phased Array	25 × 25
The kerf size The pitch size	5 μm 27 μm
Center frequency	50 MHz
Wavelength (λ)	30 μm
Input acoustic power	4.25 mW
Peak incident intensity	150 w/cm^2^
Peak incident pressure	2.15 MPa
Acoustic impedance of water	1.5 MRayls
Acoustic impedance of particle	1.4 MRayls
Speed of sound in water	1500 m/s
Speed of sound in particle viscosity coefficient of water	1450 m/s 8.9 × 10^−^^4^ Pa∙s
Density of water	1000 kg/m^3^
Density of particle	950 kg/m^3^
Diameter of particle	8λ ~ 12λ

### 3.1. Multiple-Focus Acoustic Field

**Figure 5:**
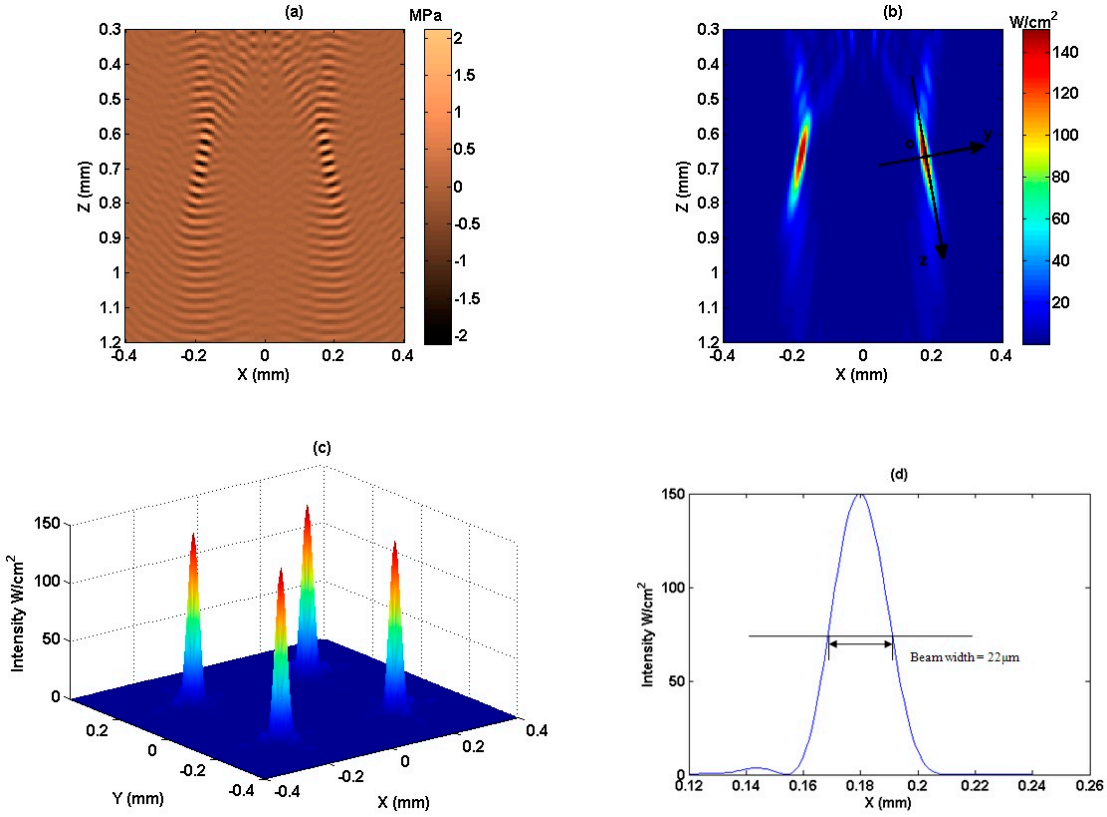
Acoustic intensity field for four focus points. (**a**) Instantaneous pressure field along the beam axis; (**b**) Acoustic intensity field along the beam axis; (**c**) Acoustic intensity field across the *z*-axis; (**d**) Intensity profile for one of the four focal points across the beam axis and passing through the focal point.

First, four focal points are created in the ultrasonic field using the pseudoinverse method to achieve the desired field patterns and intensity levels at the control points. All four control points are set as the same weight p *=* [1, 1, 1, 1]*^t^* in Equation (7) at the same depth 670 μm in the plane perpendicular with the Z direction in the XYZ coordinate system, as shown in Figure 1. By detecting the four focal points in the 3D energy field, we can get the coordinates of the four control points, which are (0.18, −0.18, 0.67) mm, (−0.18, 0.18, 0.67) mm, (−0.18, −0.18, 0.67) mm, and (0.18, 0.18, 0.67) mm, respectively. Then the pseudoinverse approach with weighting matrix described by Equation (7) is used to determine the excitation vector to synthesize the desired multiple-focus acoustic field with the maximum array excitation efficiency. After that, the fields with four focal points are obtained by combining the excitation vector with field modeling method expressed by Equations (8) and (9). The results are shown in Figure 5.

Figure 5a is the diagonal section of the instantaneous pressure field, which is through points (−0.18, −0.18, 0.67) mm and (0.18, 0.18, 0.67) mm along *z* axis. Within the instantaneous pressure field, the wave front converges to two focal points and diverges afterwards. So it satisfies the geometry and behavior of a Gaussian beam. According to Equation (9), the diagonal plane of ultrasonic intensity field with desired intensity level at control points is obtained, which is shown in Figure 5b, and used as a parameter to calculate the radiation force. Figure 5c shows the section of acoustic intensity field across the *z* axis. It can be seen that the field intensity around the four focal points is symmetrical, also the intensity at the four focal points are equal to each other. Figure 5d presents the intensity profile across the Gaussian wave propagation direction, showing the peak at the waist. It can be seen that the spatial peak intensity in the beam is 150 W/cm^2^, the beam width is defined as the spatial extent between the half power points, which is 22 μm here. Hence, the four focal points can be regarded as four beams with 22 μm beam waist.

### 3.2. Radiation Force Analysis for the Particles in Different Location

The acoustic intensity field with four multiple-focus points (Figure 5) has been created by a phased array transducer with parameters as mentioned above. It can be seen that the four focal points and the field around them are symmetry. So the radiation force in one of the beam is the same with others. Here, the directions along and across the beam propagation direction are defined as axial direction (*z*-axis), and lateral direction (*y*-axis) respectively. And the focal point *o* is set as origin of coordinates *yoz*, shown in Figure 5b. The axial and lateral radiation force are calculated in one of the four focal points following the steps indicated by the flowchart in Figure 2.

**Figure 6:**
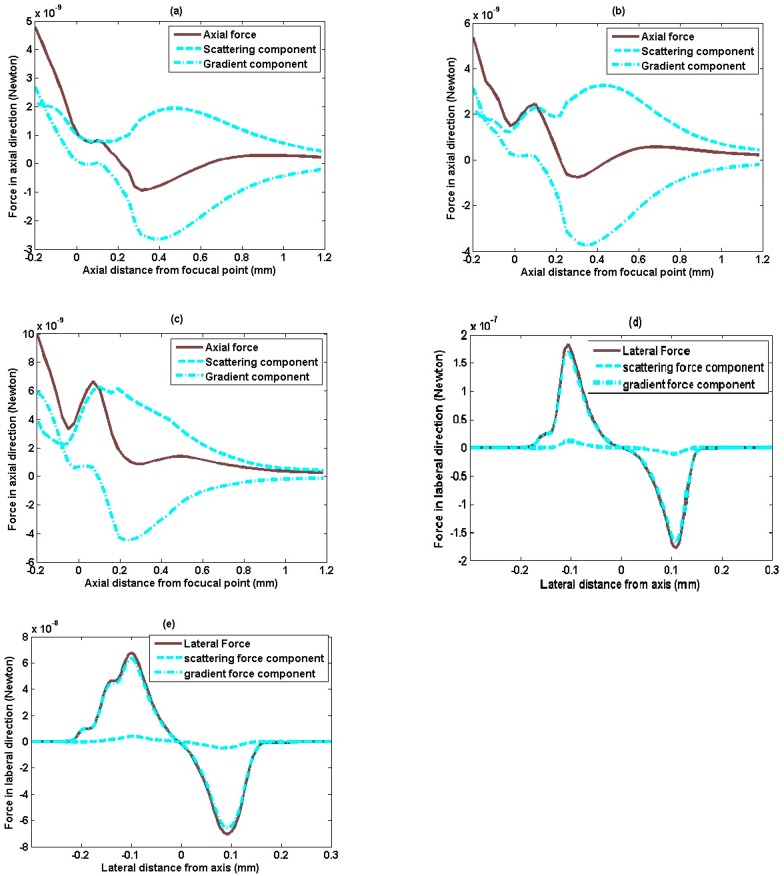
Acoustic radiation force along and across the beam propagation direction. The diameter of the particle is 240 μm in coordinate system zoy. (**a**) Axial radiation force as the particle location varies from (−0.2, 0) to (1.2, 0) mm; (**b**) Axial radiation force as the particle location varies from (−0.2, 0.02) to (1.2, 0.02) mm; (**c**) Axial radiation force as the particle location varies from (−0.2, 0.05) to (1.2, 0.05) mm;(**d**) Lateral radiation force as the particle location varies from (0.13, −0.3) to (0.13, 0.35) mm; (**e**) Lateral radiation force as the particle location varies from (0.23, −0.3) to (0.23, 0.35) mm.

In coordinate system *z*o*y* that shown in Figure 5b, the axial and lateral radiation force for a sphere with 240 μm diameter at various locations are shown in Figure 6. The axial radiation force, when the sphere moves along *z*-axis, is shown in Figure 6a. It can be seen that positive axial radiation force was first exerted to push the target particle forward, afterwards, negative axial radiation force is applied to draw particle back. Therefore, positive and negative force exists alternatively, it makes the particle be trapped. Assuming a sphere moves from (−0.2, 0.02) mm to (1.2, 0.02) mm, parallels to *z* axis, but 20 μm distance off it, the axial radiation force is plotted in Figure 6b. It is interesting to find that even its motion is off the *z*-axis, it still produces negative radiation force for acoustic trapping, though the magnitude is smaller than that on the beam axis. Figure 6c shows the axial radiation force when the particle moves along the parallel line with farther distance 50 μm off the *z*-axis. It can be seen that, although the gradient force component has similar performance with Figure 6a, the scattering force with positive value counteracts the negative force produced by intensity gradient, only the positive axial radiation force can be found in Figure 6c, which results in the failure of acoustic trapping. From Figure 6a–c, it can be deduced that the negative radiation forces along the *z* axis get smaller and smaller as increased distance off the *z*-axis. Also, the scattering force mainly contributes to the positive axial force which pushes forward, whereas gradient force gives majority negative force which attracts the objective particle back.

Figure 6d shows the lateral radiation force as the particle moves along the line that parallels to *y*-axis and passes through point (0.13, 0) mm in the *z*o*y* coordinate system. The alternative positive and negative lateral forces indicate that once the particle locates off the beam axis, the lateral force will tend to pull it back to the *z*-axis. Also, it can be seen that the dash-dot line nearly overlaps with the solid line, while the dashed line is close to the zero. Since the scattering force has the same direction with the incident ray, the major part of the scattering force is in axial direction, while the part of the scattering force for lateral force is very small. Figure 6e shows lateral radiation force as the particle moves along the line which parallels to *y*-axis and passes through point (0.23, 0) mm. Compared with Figure 6d, the results in Figure 6e show that the lateral radiation force try to keep the particle at *z*-axis similarly, however, as farther from the *y*-axis, the magnitude of the lateral force gets smaller. From the gradient and scattering forces component shown in Figure 6a–e, it can be deduced that the gradient force caused by refraction plays a much more critical role than scattering force produced by reflection in producing lateral trapping force

### 3.3. Radiation Force and Trajectory Analysis for Particles with Various Sizes

**Figure 7:**
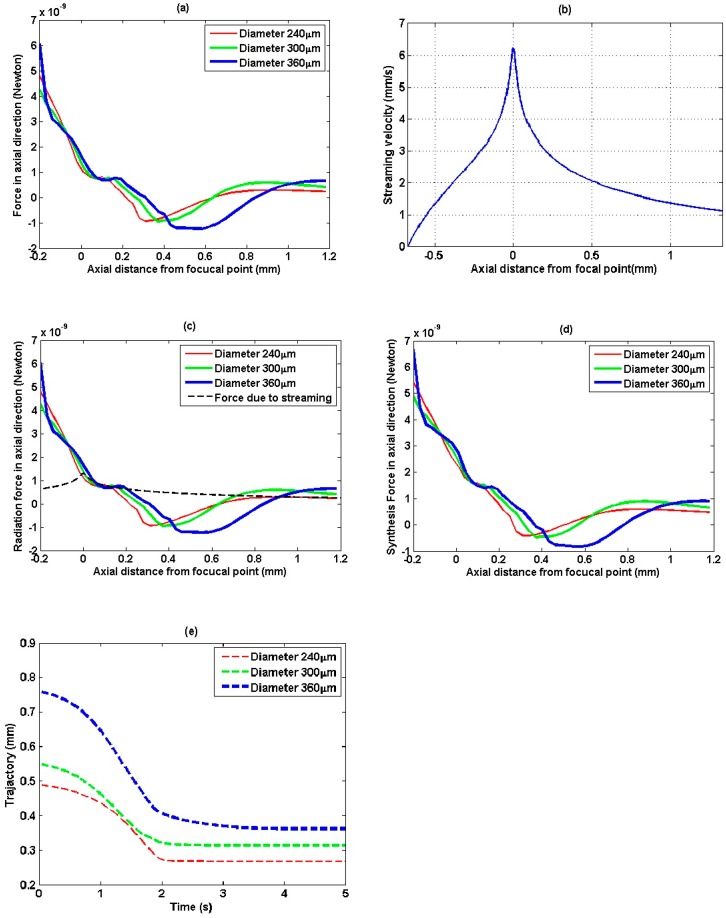
Radiation force and displacement trajectory along axial direction. (**a**) Axial radiation force as the particles travelled along axial direction or *z*-axis; (**b**) Streaming velocity along the axial direction; (**c**) Acoustic streaming velocity combined with radiation force; (**d**) Integrated force consisted with drag force and radiation force; (**e**) The plot of displacement trajectories along *z*-axis.

Particles with various diameters are also used to evaluate the trapping performance. Meantime, according to Newton’s second law of motion, the displacement trajectories of particles are determined after motion analysis in consideration of radiation force and the resistance of water viscosity. To simplify the analysis, the radiation force and displacement trajectory by axial and lateral direction are calculated separately. Figure 7a shows the axial radiation force of particles with varied diameter from 180 μm to 360 μm traveling along the *z*-axis in the coordinate system *z*o*y*. It is intriguing to note that bigger trapping force and longer attractive radiation force region are more likely to occur when using bigger particles.

To investigate the acoustic streaming effect on multiple-trapping acoustic tweezers, we introduce the approach studied by Nowicki [25] to estimate the axial streaming velocities. The streaming velocity along the axial direction is shown in Figure 7b. The result of drag force due to acoustic streaming velocity combined with radiation force along the axial direction is shown in Figure 7c. It can be noticed that the drag force is positive when the radiation force is negative in the trapping region, it means drag force intends to prevent drawing back target particle towards trapping point. The integrated force consisted with drag force due to acoustic streaming and radiation force along the axial direction is shown in Figure 7d. We can see even consider the acoustic streaming effect the integrated force along the axial direction is still negative in the trapping region, which means the particles can be trapped in this region.

**Figure 8:**
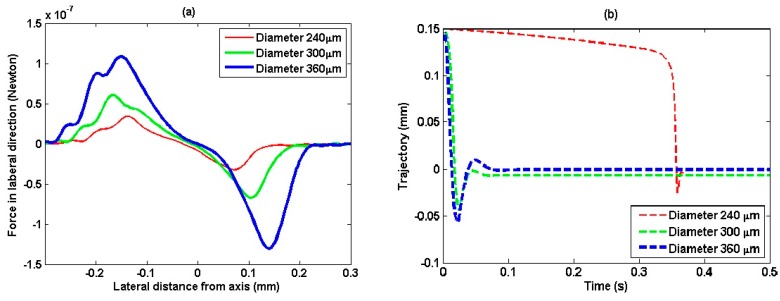
The radiation force and displacement trajectory across axial direction. (**a**) Lateral radiation force as they travelled across axial direction; (**b**) Displacement trajectory of the particles with various diameters across axial direction.

Figure 7e shows the plot of displacement trajectories along *z*-axis, which is calculated with the consideration of axial radiation force and the resistance of water viscosity. The initial speed is zero and the initial position is in the end of negative force points in Figure 7d. It is noted that although the initial positions are different, the particles are generally pulled back and finally captured within four seconds at their balanced positions. Assuming the particles are located on the lines passing through balanced positions in *z*-axis and paralleling to *y*-axis, and their locations varying along this line, the lateral force along this line could be calculated and is plotted in Figure 8a.

It can be noted that although the asymmetric lateral radiation force at the two sides of *z*-axis, the lateral radiation forces have opposite directions at two sides of *z*-axis, which means that once the particle is off *z*-axis, lateral radiation force will tend to trap it back.

Since there is almost zero radial acoustic streaming velocity at the ultrasonic transducer center, the drag force due to radial acoustic streaming effect is omitted when calculate the trajectory as particle move across the axial direction. Figure 8b shows the particles displacement trajectory along this line, which are calculated with the consideration of lateral radiation force and the resistance of water viscosity. The initial lateral positions are 150 μm off the beam axis and the initial speed are zero. It reveals that all the three particles can be trapped near the *z*-axis.

Given the specific particle size, the maximum axial trapping force, its axial trapping position, axial trapping regions and maximum lateral trapping force are listed in Table 2. It can be seen that the axial and lateral trapping force gets bigger when the size of particle increases. The magnitude of lateral force is much bigger than axial force, which indicates that the lateral trapping is easier to be achieved than the axial trapping. It also shows that the axial trapping range increases as the increased size of the particles.

**Table 2:** Summarized results for particles of various size.

Particle Diameter (μm)	Max. Axial Trapping Force (10^−^^10^ N)	Max. Axial Trapping Position (μm)	Axial Trapping Region (μm)	Max. Lateral Trapping Force (10^−^^10^ N)
240	−9.3	490	222	338
300	−9.5	550	235	606
360	−12.3	760	400	1087

## 4. Conclusions

A multiple trapping acoustic tweezers model is proposed in this paper. The pseudoinverse approach is used to produce desired intensity levels at the control points. Then, the pressure and intensity at any field point can be obtained by modeling the surface of the phased array. Besides, scattering force and gradient force at various positions are also evaluated to analysis their relative components to the effect of the acoustic tweezers. The radiation forces along and across the beam propagation direction for particles with various diameter and positions are calculated based on the acoustic ray approach. In addition, this paper reveals the trapping trajectory along and across the beam propagation directions with the consideration of acoustic streaming effect and the resistance due to viscosity of water. The results obtained demonstrate that the acoustic tweezers have feasibility for multiple trapping in both the axial and lateral directions. They could be used for non-contact and non-invasive manipulation of small particles such as cell sorting, trapping, and manipulation. Cancerous cells could be separated from healthy cells. The measurement of mechanical properties of the cell could also be achieved by acoustic tweezers by non-contact squeezing the cell. In addition, they could be used for drug delivery to drive particles along and across the wave propagation direction.
